# Supplementary material for: The Quantitative-Phase Dynamics of Apoptosis and Lytic Cell Death
Source: Sci Rep. 2020 Jan 31;10:1566. doi: 10.1038/s41598-020-58474-w (PMC6994697; doi:10.1038/s41598-020-58474-w)
Supplement: Supplementary file 2 — Supplementary information2 [file 41598_2020_58474_MOESM2_ESM.docx]

Description of additional supplementary files

The Quantitative-Phase Dynamics of Apoptosis and Lytic Cell Death

Tomas Vicar, Martina Raudenska, Jaromir Gumulec, and Jan Balvan

**Supplementary video 1:** **Quantitative phase time-lapse of cell dying by apoptosis (characteristic by high cell density, high cell dynamic score).** DU-145 cells exposed to 0.1 μM doxorubicin. Vertical red line indicate membrane rupture. 10x magnification. CDS, cell dynamic score; QPI, quantitative phase image; PI, propidium iodide.

**Supplementary video 2:** **Quantitative phase time-lapse of cell dying by lytic cell death (characteristic by low cell density, low cell dynamic score).** DU-145 cells exposed to 0.1 μM doxorubicin. Vertical red line indicate membrane rupture. 10x magnification. CDS, cell dynamic score; QPI, quantitative phase image; PI, propidium iodide.
